# Practising in a pandemic: A real time study of primary care practitioners' experience of working through the first year of COVID-19

**DOI:** 10.3389/fsoc.2022.959222

**Published:** 2022-10-06

**Authors:** Emily Burn, Judith Smith, Rebecca Fisher, Louise Locock, Kirsty Shires

**Affiliations:** ^1^Health Services Management Centre, University of Birmingham, Birmingham, United Kingdom; ^2^The Health Foundation, London, United Kingdom; ^3^Heath Services Research Unit, University of Aberdeen, Aberdeen, United Kingdom; ^4^Moss Grove Surgery, Kingswinford, United Kingdom

**Keywords:** general practice (MeSH), COVID-19, rapid research methods, audio diary method, interview (MeSH)

## Abstract

This article presents reflections on the lessons learnt from developing and initiating a rapid research project in 4 weeks during the first year of the COVID-19 pandemic. The article highlights the importance of selecting methods appropriate to rapid research, discusses the challenges of data collection in a shifting context, and the importance of the research team being prepared to cede some degree of control over the data collection process. To protect staff and patients and prevent the spread of COVID-19, general practice shifted to remote service delivery and consultations occurred via the telephone or online platforms. In the study, submissions were collected from those working in general practice to capture their experiences of the first year of the COVID-19 pandemic. Participants could choose how to submit their narratives, with some preferring to be interviewed and others contributing self-recorded submissions. This article offers practical reflections in response to the challenges of carrying out rapid research during a pandemic, including the importance of constructing a research team which can respond to the demands of the study, as well as the benefits of an expedited ethical review process. The study highlighted the importance of selecting appropriate methods to facilitate the rapid collection of data. In particular, the authors reflect on the differences between participants' response to interviews, written submissions, and audio diaries. Open approaches to data collection were found to encourage participation and reflexivity and also generated rich narrative accounts. Rapid research has progressed our understanding of general practice's experience of the first year of COVID-19.

## Introduction

There is no clear definition or criteria informing when a project can be considered “rapid” research. The literature does acknowledge that rapid research can be defined in terms of the timescale of the project (Vindrola-Padros and Vindrola-Padros, 2018), including the time taken to establish, or complete the project, as well as the design of the project which may incorporate longer evaluations and include early and ongoing reporting, learning and feedback (McNall and Foster-Fishman, 2007). In this article, we will discuss some of the lessons gained from mobilizing and managing a research project within the dynamic context of the first year of the COVID-19 pandemic. The COVID-19 pandemic presented exceptional challenges to the delivery of health services. In the United Kingdom (UK) and elsewhere, general practice had to radically reorganize service delivery as consultations shifted from face-to-face interactions to telephone calls or via online platforms. Furthermore, hot hubs were established to treat patients who had COVID-19 symptoms, as well as new staff and roles added to general practice teams.

This article explores the challenges of establishing a UK-based project exploring the response of general practice to the COVID-19 pandemic. Our study started in early spring 2020 and captured narratives from the general practice workforce throughout the first year of the COVID-19 pandemic. With support from the Health Foundation, we collected submissions from general practitioners, practice nurses, and practice mangers, aiming to capture a range of experiences of the COVID-19 pandemic. In this article, we consider the challenges of carrying out rapid research during a pandemic when the project was designed and initiated within 4 weeks. We will first summarize the study we undertook as an example of rapid research due to the limited timeframe available to establish the project. The article then considers how the project was designed to respond nimbly to shifts in policy across the first year of the pandemic, in addition to the challenges presented by longitudinal data collection and analysis. We also reflect on the practical considerations as to how we ensured participants could share their contributions swiftly and securely, while meeting ethical review requirements.

From a focus on the practicalities of undertaking rapid research, we then discuss how we as researchers experienced working on the project. We will consider how the composition of the research team assisted us to reflect the changing policy context within the project. Our data collection techniques were open, flexible, and gave participants the space to decide when and how they would like to contribute their submissions. We found that self-directed submissions afforded participants room to discuss the challenges faced by the general practice workforce during the pandemic. Our approach required us to cede an element of control of the project to those who were narrating their experiences. We consider the difficulties and opportunities this presented for the project, including a reflection on the depth and richness of the narrative accounts shared with us, and the approach taken to curating and analyzing these. The article will conclude by exploring participants' experience of contributing narratives and drawing out the different aspects of reflexivity which defined and enriched this study. The article builds on the literature on rapid research by exploring the tensions that can arise when a project is time-sensitive and discusses the benefits of open research methods to the recruitment and retention of participants, along with the depth of participants' submissions.

## Exploring the shifts within general practice during the first year of COVID-19: The need for rapid research

As noted above, there are varying definitions of what timeframes can be considered rapid. In a systematic review of rapid research methods during complex health emergencies, Johnson and Vindrola-Padros (2017) exclude studies where data collection went on for longer than 6 months. However, rapid research may also be defined *via* the intention behind the research to inform the ongoing development of policy or interventions and some designs may be longer and feedback initial findings (Vindrola-Padros et al., 2020b, p. 2193). Focused ethnographies are an example of rapid research and are a “condensed alternative” (Locock et al., 2020, p. 19) compared to more time-intensive conventional forms of ethnography. This form of ethnography is distinguished by the pre-selection of the topic under study which occurs within a specified context (Higginbottom et al., 2013; Stahlke Wall, 2015). Data collection within focused ethnography occurs over a shorter time frame and can be intense compared to more traditional conceptualizations of ethnography (Knoblauch, 2005).

We consider our project to be an example of rapid research due to the speed by which it was established and its rapid iteration cycles. It took 4 weeks from initial conceptualization to data collection. In this time, the project team was formed, ethical approval secured, and participants recruited to ensure that the project could respond to the rapidly escalating impact of the COVID-19 pandemic in the UK. Fieldwork for our project ran for 11 months, with the first narrative accounts collected in April 2020 and the last collected in March 2021. At the start of the project, we were not sure how long we would need to collect data given the great uncertainty associated with the COVID-19 pandemic. Approaches to the management of COVID-19 evolved over time and we needed to capture these shifts within our project. Therefore, we invited participants to submit narratives across the first year of the pandemic. Rapid research is not synonymous with a lack of depth, although researchers do need to be aware of measures that can be taken to strengthen rapid research (as explored by McNall and Foster-Fishman, 2007). While the rigor of the research design is paramount, there is a balance to be struck between the scope of the research and expectations as to the delivery of findings (often determined by funding availability). We explore this balance by discussing the challenges of establishing rapid research, along with considering the opportunities presented to researchers to pursue an open research design in which participants can choose the way they wish to contribute to the research. To progress these points, we first outline how general practice responded to the COVID-19 pandemic.

At the start of the COVID-19 pandemic, general practice shifted to remote consultations wherever possible and face-to-face consultations were offered only when deemed necessary, furthermore non-urgent elective operations were postponed initially for 3 months (NHS England Improvement, 2020a). There was also highly negative reporting on general practice from some sections of the UK media, as erroneous narratives about general practice being “closed” and GPs being unprepared to offer face to face consultations were reported (Mroz et al., 2021). A letter sent from NHS England Improvement (2020b) which emphasized the importance of providing face-to-face appointments and noted that communications from practices should not suggest that they were closed compounded such inaccurate messages. General practice was also involved in delivering the COVID-19 vaccination programme (introduced in December 2020) to their local communities. As the pandemic evolved and policies to attempt to manage the pandemic shifted, including the introduction, easing and subsequent reintroduction of lockdowns, we saw the benefit of continuing data collection to capture the thoughts and experiences of members of the general practice workforce. By establishing the project at speed, we were able to capture the experiences of the general practice workforce in real time from the start of the pandemic and throughout the first year—reflecting not only the changes within the delivery of general practice services but also how participants experienced these changes at both a personal and professional level.

## Summary of the study undertaken

A purposive sampling approach (Blaikie, 2009) was used to recruit participants to capture a range of experiences. The study recruited a mixed sample that incorporated a spread of geographical locations and levels of experience, including salaried and partnered general practitioners. Salaried general practitioners are employed by their practice, whereas practice partners have a greater involvement in setting the direction of the practice. In total, 17 participants were recruited, with 13 general practitioners, 2 practice nurses and 2 practice managers contributing submissions.

Decisions on the clinical management and containment of COVID-19 evolved across the first year of the pandemic (Health Foundation, 2021). It was important that the study captured participants' responses to these changes. Open questions were devised and revised by the research team to reflect the evolution of the pandemic. Participants were not obliged to respond to these questions and were invited to provide a personal account of their experiences and highlight the most pressing issues of concern. Participants could choose the method in which they submitted their accounts. While some participants submitted accounts *via* written accounts or voice notes, others preferred to be interviewed either by telephone or by using an online platform. The research team sent batches of questions to participants at six points throughout the year. Interviews were audio recorded and interviews and voice notes were professionally transcribed, and were loaded onto NVivo version 12 (QSR International Pty Ltd, 2018). Thematic coding (Braun et al., 2018) was carried out by one member of the research team throughout the data collection process. The research team met frequently to develop the coding frame, discuss emerging themes, and develop the questions posed to participants as suggestions to guide their narrative accounts. Once data collection was completed, the research team developed an overview of the themes which explored changes and continuities throughout the evolution of the pandemic.

Participants' submissions described the great speed at which changes were made to the delivery of general practice services. As we explore in Burn et al. (2021), participants' submissions at the start of the COVID-19 pandemic often discussed a sense of uncertainty. This uncertainty had a clinical dimension, in terms of how to respond to COVID-19, the effect of the pandemic on existing health inequalities and the ongoing relationship between primary and secondary care. Furthermore, some general practitioner participants reflected on the uncertainty they were experiencing with regards to their professional identity and how the rapid and widespread adoption of remote consultation (felt by some to be transactional) might influence how they relate to their role. As the pandemic progressed, some participants discussed how the pressures of social distancing had led to strained relationships with patients. Participants' submissions offered reflections on the exhaustion and burnout experienced across general practice. While some participants' submissions noted the potential positive long-term changes to service delivery, their submissions reflected a continuation of the strain experienced by the general practice workforce pre-pandemic.

## Lessons in developing rapid research

Our project's longitudinal design captured participants' real-time reflections on an ever-changing and unpredictable environment where attempts to manage COVID-19 were introduced and then refined. We build on work exploring the experiences of healthcare professionals to the pandemic (Vindrola-Padros et al., 2020a; Borek et al., 2022) by focusing solely on the experiences of general practice. While the high levels of fatigue and stress experienced by the general practice workforce has been explored within previous research (Di Monte et al., 2020; Sharma et al., 2020; Trivedi et al., 2020; Xu et al., 2020; Sotomayor-Castillo et al., 2021), often these studies use a cross-sectional quantitative research design.

The rapid approach of this study ensured that data were gathered from the start of the pandemic and allowed comparisons to be drawn continually throughout the period of data collection. By continuing to collect submissions throughout the first year of the pandemic, we were able to capture participants' responses to the evolving COVID-19 situation. Doing so facilitated a greater depth to the exploration of uncertainty and participants' reflections on their profession, and acknowledged the shifts in participants' perceptions over time. Robust project management supported the recruitment of participants and associated data collection.

Reflecting on the research team's experience of developing and administering the project identifies a series of lessons which may be useful for future rapid research projects. The discussion will first consider the lessons the research team gained when establishing the project and will reflect on the composition of the research team, as well as the benefits of an expedited ethical review process. These discussions reflect the practical considerations of which researchers engaged in rapid research studies should be aware. The discussion then reflects on the research design and the way in which open research methods can facilitate reflexivity from participants.

### Constructing a research team: The importance of professional networks

The quick formation of a research team is important when undertaking rapid research. In our experience, creating a research team was dependent on professional networks and pre-existing relationships. The project was born out of an ongoing Twitter conversation between two of the authors (JS and LL). As the idea progressed, a research team was formed by one member of the team (JS) and comprised of four people across three institutions. Three members of the team have a non-clinical background and one has a clinical background as a general practitioner. The small team aided communication throughout the design and administration of the project and ensured that decisions could be made swiftly.

Collaborating with a clinician meant that we were able to benefit from in-depth policy knowledge and support with recruitment through access to networks of the general practice workforce (as also noted by Chew-Graham et al., 2002 and Patel et al., 2017). The involvement of a general practitioner within the research team assisted the process of analysis and interpretation as they were able to provide a sense check of initial interpretations as someone with clinical experience during the COVID-19 pandemic. Within our project, interviews were completed by non-clinician members of the team. As discussed by Chew-Graham et al. (2002), interviews with expert professionals can be influenced by an interviewer's identity. If the researcher and participant do not have a shared experience, the interview can avoid falling on shared assumptions and lead to greater explication and a more developed depth of data. Furthermore, Coar and Sim (2006) note that when the interviewer and participant share a professional background the participant may perceive the interview as a test of their professional standing and identity which may inhibit the level of detail in their response. The shared identity between the researcher and participant may also mean that there is a sense of “professional cooperation and solidarity” (Coar and Sim, 2006, p. 254) and a more trusting relationship may develop between interviewer and participant. Nevertheless, we found that interview participants still discussed their experience of the first year of the COVID-19 pandemic in great depth despite having non-clinician members of the team conduct interviews.

When establishing our rapid research project, one member's pre-existing working relationships meant that a research team could be formed quickly and a clear division of labor created with each member contributing to different elements of the project. The disruption of COVID-19 led to the suspension of other areas of work and one member of the team (EB) was able to act as a central point of contact and co-ordination across the project. While the disturbance created by COVID-19 is exceptional, our experience highlights the importance of project management and having someone tasked with coordinating the team and ensuring that deadlines are met. Our opportunistic approach to forming a research team worked well for this project; however, we may have benefitted from having more time to build a wider team. Those initiating a rapid research project are unlikely to have this luxury of time—demonstrating the benefit of researchers developing an extensive professional network.

### Ethical review: An expedited process

Gaining ethical approval has often been noted as a barrier to rapid research (McDonach et al., 2009; Vindrola-Padros et al., 2020a,b). However, we benefitted from an expedited ethical review process when working to establish the project. Research projects exploring aspects of COVID-19 and its management were subject to a fast-tracked ethical review process at the university which removed the bureaucratic delays and competing demands that can often affect projects. Nevertheless, in a systematic review of rapid ethnographies in healthcare organizations, Vindrola-Padros and Vindrola-Padros (2018) found that none of the included studies discussed delays generated by ethical governance processes. The authors of the review question whether ethical review committees are becoming more aware of the time pressures related to undertaking rapid research and suggest this as an area for future study. Research within the National Health Service (NHS) requires approval from the Health Research Authority (HRA), the body overseeing the regulation of different elements of health and social care research (HRA, 2022b). This additional level of approval can create a further (although understandable) complexity when establishing a rapid research project. There is a decision tool (HRA, 2022a) which can be used to identify projects which require approval from the HRA. Our project did not need ethical approval from the HRA as participants were not recruited *via* NHS channels. Furthermore, participants were asked to volunteer their own time rather than participate during working hours to avoid burdening the NHS. Consequently, our project was only required to gain ethical approval from the University of Birmingham, and we benefitted from COVID-19 research projects being prioritized throughout the ethical review process. Researchers are dependent on the ethical review process and there is not much the research team can do to accelerate their project gaining approval. However, there is the opportunity to consider how these expedited processes can be maintained after the COVID-19 pandemic (Vindrola-Padros et al., 2020a).

### Recruiting participants: Making the most of pre-existing contacts

Participants had to be recruited quickly to the study to ensure timely data collection. It was important that we gathered a broad overview of the experience of the general practice workforce and so we aimed to recruit not only general practitioners, but also practice nurses and practice managers. We used a purposive sample (Blaikie, 2009) to recruit participants from a range of general practice roles across geographical locations and different levels of experience. We found it helpful to map the research team's network to identify potential sources to recruit participants and a small number of participants were known in a professional capacity to the research team. One source of recruitment was Next Generation GP, a leadership programme and network for emerging GP leaders (Next Generation GP, 2022). The research team sent an introductory email to potential participants establishing the study and providing information as to how to get involved. The project was also promoted on Twitter which generated some expressions of interest. Some potential participants offered to send out the invitation to their own network as a form of snowball sampling and the invitation was included in a staff newsletter for a large general practice partnership.

Thinking about how participants will interact with a rapid research study can support recruitment. The research team were aware of the time pressures on potential participants and so communications introducing the project highlighted the control participants would have as to when to contribute submissions and aimed to alleviate any perceived research burden. Furthermore, the research team avoided setting a hard deadline in which participants had to be recruited. Instead, our rolling approach meant that recruitment to the project gained momentum as word was spread about the project. Still, the research team found that we received a small number of expressions of interest in the project which were not converted into full participation—something that reflects the great deal of strain the general practice workforce was (and continues to be) under.

In the early days of the pandemic, there was still considerable uncertainty about remote recording and file transfer. The short timeframe available to establish the project meant that the research team had to work with colleagues in the University's IT department to find sometimes sub-optimal solutions to allow these, often large, audio files to be transferred. We settled on using the University's file hosting service facility which allowed files to be transferred securely and meet information governance requirements. Since then, experience of doing remote qualitative research has generated separate areas of learning (Gratton et al., 2020; Richardson et al., 2021). Communicating the technical requirements of remote participation to study contributors is important—particularly given the time demands of rapid research and wider pressures on participants.

### Research design—Encouraging participation and reflexivity

The research design had to respond to the two related aims of the project to capture both 1. responses to the external policy environment, as well as 2. participants' internal states and their reaction to the wide-ranging pressures of COVID-19 on both personal and professional lives. The research team found that an open research design facilitated responsive data collection which could capture shifts in attempts to manage COVID-19. The approach allowed the research team to collect data in real-time and reflected the experience of what it was like to work in general practice during the pandemic, the changes to service-delivery, and the challenges associated with this time-period.

The research methods within rapid research should take account of participants' circumstances. Participation should be made as easy as possible to maintain engagement with the project—particularly when rapid research has a longitudinal element. The increased demands placed on participants during the pandemic meant that the research design had to incorporate a degree of flexibility and allow participants to contribute submissions easily, without placing too much demand on their time. Participants could choose the method that they used to contribute their submission. Ensuring participants could submit their accounts using a range of approaches seemed to work well to encourage participation as 13 of the 17 participants contributed multiple submissions. Some participants used different methods to submit their contributions (for example, written contributions for initial submissions and interviews with the research team as the study progressed). In addition to making participation as easy as possible, it was important that the research team's approach to data collection was not overly prescriptive. An open approach to data collection gave scope for the project to uncover previously hidden accounts that may have been missed were a more directive approach used.

The research methods used to collect submissions all facilitated participant reflexivity. Reflexivity refers to the ability of individuals to consider their own feelings, perceptions and motives and the influence this may have on how they respond in each situation (Archer, 2007). The project was interested in exploring accounts of professional identities in general practice and whether COVID-19 affected how people related to these identities. Identities inform how individuals interpret the social world and comprise the characteristics and roles which inform how individuals define themselves (Oyserman et al., 2012, p. 69). Participants' reflexivity enhanced simple descriptions of the experience of working in general practice during the first year of the pandemic to provide developed accounts which consider how the participant relates to the experience—for example their emotional responses and their motivation for taking particular courses of action. Encouraging reflexivity can benefit rapid research which prioritizes developing a depth of understanding of people's experiences.

There were some differences in how participants interacted with audio diaries compared to written submissions. Perhaps reflecting the immense challenges placed on general practice, written submissions were often relatively brief and were less detailed compared to audio-diaries, a difference also found by Hislop et al. (2005). Despite the comparative brevity of some of the written responses, participants still reflected on their experiences of the changes brought about by the COVID-19 pandemic and some participants did contribute more personal reflections on the difficulties visited by the pandemic on their home life, as well as their concerns and hopes for the future of general practice. By way of contrast, participants' audio-diaries promoted self-talk (Crozier and Cassell, 2016), in other words participants' inner monologs and facilitated in the moment reflection from participants on their experiences (Monrouxe, 2009; Williamson et al., 2015; Dangeni et al., 2021) which can facilitate participants to construct a sense of their identities (Verma, 2020, 2021). The flexibility of audio diaries also allows participants time to reflect and this approach does not require instant responses as can be the case in interviews (Crozier and Cassell, 2016).

Compared to audio diaries, interviews are less flexible for participants as they need to be scheduled. However, the presence of the researcher did have advantages which were particularly beneficial given the demands of rapid research. An advantage of semi-structured interviews over audio diaries is that the interviewer can confirm their understanding with participants, as well as probing on further points of interest (as also noted by Cottingham and Erickson, 2020). While the participant can still direct the conversation in semi-structured interviews, there is a greater reliance on the interviewer to draw out and co-construct reflections from participants (McGrath et al., 2019). The interviews with participants were guided by a discussion guide that incorporated the open questions intermittently posed to participants. Within the interviews there was a tendency for participants to first cover the substantive changes that had been made to service delivery (as identified through questions such as “*What changes have been made in your practice since the start of the COVID pandemic?”*) before then progressing to discuss more personal responses (as encouraged by the question “*How is this affecting you personally in the context of the rest of your life?”*). From the perspective of the researcher (EB) who undertook most of the research interviews, it felt as though there could be an almost jarring shift from discussing the substantive changes made to service delivery towards the discussion of more personal, or what could potentially be sensitive, topics. Aware of this shift in tone in the interview guide, there was a tendency for the researcher to check-in with the participant that they were comfortable to discuss how they were personally responding to the pandemic.

Within submissions *via* audio diaries, the relationship between researcher and participant is more distant, however, participants' contributions still reflected a sense that their account would be heard by the research team. Participants often opened by introducing themselves, with some reflecting on the last time they had contributed a submission:

*This is [NAME] recording on the 4th of September, for the narrative accounts on primary care practitioners in the time of COVID project. So firstly, it's been a while since my last recording. Sorry about that. It kind of slipped my mind and maybe that is symptomatic of the difficult summer that we've had. Participant 7*

During interviews the researcher can ask for clarification or to go back and ask for more information on a particular topic raised by the participant (Bowling, 2014). During interviews there were examples of uncertainty from participants, as demonstrated by participant 14's comment “*that's not really answered your question, sorry”* while other participants asked for confirmation at the end of the conversation that the interview had been helpful. At the start of the pandemic, participants' submissions had more ground to cover as changes to service-delivery were discussed before then reflecting on personal reactions to these changes. As the pandemic evolved, submissions had a broader focus and were less directed to changes to service-delivery and discussed the pandemic more generally with a focus on the effects of the pandemic on the profession.

Throughout our study, participants were not asked directly about their experience of how they found participating in the project. However, one participant (Dr Kirsty Shires, a practising GP) shared their thoughts and experience of being involved in the study. Considering participants' own reflections on their involvement with the project can further our understanding of how participation in a research project can contribute to participants' meaning-making (Cassell et al., 2020). Asking participants to share their experiences of being involved in the project may enhance rapid research as responses can add depth to the findings by indicating how self-understanding had been developed through participating in the project.

As seen in the summary below, the participant highlights how self-recording their submissions offered a respite from the written word at a time when they had to read and respond to high quantities of information. For this participant, the decisions made about the design of the research eased and encouraged participation. Furthermore, the participant notes an altruistic motivation by contributing to a project that has recorded the experiences of the general practice workforce during a historic time. Such reflections highlight that the general practice workforce may have a range of motivations informing the decision to become involved in a research project (as explored by Gunn et al., 2008; Brodaty et al., 2013 and Patel et al., 2017). Thinking about these motivations may inform different approaches to encourage participation in research projects—something particularly useful when a project needs to be established in a limited amount of time.

### Practising in a pandemic: Reflecting on my experience of contributing to the narrative accounts study—Dr Kirsty Shires

I got involved in the narrative accounts study in June 2020, after reading about it on Twitter. I worked both in general practice as a salaried GP and in medical education. Like most people across the UK and indeed the globe, the COVID-19 pandemic had a profound effect on my working life. In the GP setting, I was still working physically in the surgery, but in my medical education role my team and I were all working from home. We also had to make decisions about medical student placements and how to continue these remotely, which involved regular communications with teaching practices.

Providing submissions to the narrative accounts study enabled me to have some reflective space to process the many changes that were occurring. There was so much information coming from different sources, the pathophysiology of this new virus was becoming clearer, the impact on the general population was extraordinary. The transformations in secondary care were televised and reported in the media, and I felt for my colleagues witnessing overwhelming sickness and death. Using voice recordings was a novel way for me to reflect, but I found it to be a relief not to have to write my thoughts down—there was so much written information both to read and to issue to others. The flexibility of the type of submission that could be uploaded was very considerate to the pressure contributors were likely to be under. The periodic reminder email with some prompt questions provided a helpful framework and it was easy to share the recordings on the secure platform. Later, there was an opportunity for a virtual interview; the dialogue and human interaction helped make sense of the situation. I also submitted an example of some information I wrote for the practice website and a rather clumsy poem.

It might sound grandiose but I did have a sense that we were living through a historic time. Having contemporaneous first-hand accounts I thought could be important for looking back on these events and for understanding and learning from them. Not everyone could be a hero and save lives on intensive care units, so this at least felt like a small contribution I could make. It has been a privilege to be involved.

## Rapid research and the benefit of an open research design

During the study, the participants were facing a uniquely challenging time in trying to orientate their clinical practice towards COVID-19, but also reflect on the impact of the pandemic on their understanding of their chosen profession. It was necessary for our project to be set-up rapidly, along with being built on methods that would facilitate rapid data collection. Given the demands of the first year of the pandemic, the research design had to give participants the space to choose the most appropriate way to respond to requests for submissions. There is a tendency for qualitative research to emphasize the value of face-to-face interviews as a way of encouraging participants to explore their thoughts, beliefs and (in)actions and the meanings attached to these (for example, Way et al., 2015 discuss the contribution of dialogic interviewing to promote self-reflexivity). However, the demands of rapid research means that the speed and flexibility of method need to be prioritized, while also ensuring the rigor of the research. The open approach within the project facilitated rapid research, however this did necessitate the research team ceding some degree of control over data collection. Self-recorded and written submissions were directed by the participant. While the research team sent out prompts, to some extent the team had to wait to receive submissions and were uncertain as to the level of detail that would be found within participants' accounts. Research is rarely a linear process (Morse et al., 2002), and every project is likely to encounter its own difficulties and unexpected obstacles (Clark, 2007). Nevertheless, our experience of rapid research and open research methods engendered a sense of almost passivity unusual to researchers during data collection. As the project progressed, there was a growing sense of ease that this open approach would be successful in securing contributions. However, researchers using an open approach to data collection that is conducive to rapid research should be comfortable with giving up an element of control over the research process.

Furthermore, the open and participant-led methods of data collection also facilitated reflexivity within participants' responses. Our use of the term reflexivity refers to the processes by which people consider and reflect on their situation and actions, however, as discussed by Doyle (2013) there are nuances in the conceptualization of reflexivity. Yang (2015) discusses a tendency for reflexivity to be conceptualized *via* the experience of the researcher rather than the participant. Cassell et al.'s (2020) use of the term participant reflexivity reflects on the potential for involvement in research to influence the reflexive thinking of participants and note the lack of attention this occurrence has received in the methodological literature. As discussed, time constraints can be a challenge within rapid research. An open research design can respond to these challenges by allowing participants the option to choose the most appropriate method to contribute to the project. Continued participation can be encouraged as a result. Furthermore, the open research design still facilitated reflexivity and led to depth and richness within the data collected. Exploring participants' reflexivity and the way people “engage in self-interrogation and reflection” (Way et al., 2015, p. 723) further develops the case for those leading rapid research to consider the merits of an open research design.

Reflecting on a project exploring work-life balance, Cassell et al. (2020, pp. 758–761) identify different forms of reflexive dialogue participants engage in when discussing their participation in the study. These forms of participant reflexivity include: (1) constructing a self-narrative, (2) challenging taken for granted assumptions, (3) emotional dialogue, and (4) action dialogue. When discussing the benefit of an open research design within rapid research, we use the term reflexivity as a more general account of self-reflexivity, rather than participants' insights gained through being involved in a research study. The emphasis within our analysis is on the way individuals relate to their own contexts, thoughts, feelings, and actions, and reflects how the term is used in wider debates on the conceptualization of agency (Archer, 2007; Akram, 2019). This conceptualization is in contrast to Cassell et al.'s discussion which emphasizes participants' reflections on their experience of contributing to a research study. Despite this distinction, the work of Cassell et al. (2020) is helpful in clarifying different expressions of reflexivity.

Applying Cassell et al.'s forms of participant reflexivity to the data collected within our study is valuable as it identifies the multiple dimensions to reflexivity which can be encouraged through an open research design. In Table 1, the different expressions of reflexivity are outlined, along with examples of where these dimensions were found within the narratives collected from the general practice workforce. This mapping across participants' narratives indicates that giving participants the choice as to how they wanted to engage with the project facilitated processes of reflexivity and resulted in richer data, rather than a simple account of the shifts in service provision within general practice. The project's findings had a greater depth as a result. Rapid research designs should consider whether promoting reflexivity is appropriate for the aims of the research and how open approaches to data collection could facilitate reflexivity.

**Table 1:** Examples of reflexive dialogues.

**Reflexive dialogue (adapted from Cassell et al., 2020, pp. 758–761)**	**Definition**	**Examples within general practice narratives**
Constructing a self-narrative	Participants' self-awareness and account of who they “are” as a member of the general practice workforce	Participants' discussions of their professional identity were often rooted in the importance of the quality of relationships with their patients
Challenging taken for granted assumptions	Participants' awareness of the assumptions that inform their understanding of general practice	Accounts of general practice were grounded in relational care. However, there was the potential for this understanding to be challenged by the move to remote consulting which could feel transactional
Emotional dialogue	Reflections on the emotions generated when considering the pandemic at both a personal and professional level	Uncertainty generated by the early stages of the pandemic. Two forms of uncertainty were identified: clinical uncertainty and how service change may affect the future of general practice
Action dialogue	Reflexivity informing the actions of participants in response to the pandemic	Participants' actions to support a sense of work-life balance and the threats to coping mechanisms presented by social distancing measures

## Study limitations

Despite the study providing an insight into the experience of the general practice workforce, it was affected by a number of limitations. The open approach to data collection was central to the success of the project as it facilitated participants choosing the method that was most appropriate to their situation. While frequent invitations to submit accounts were sent to participants, not all participants responded to these invitations. This occasional lack of response is perhaps to be expected due to the challenges presented during the first year of the COVID-19 pandemic or may reflect that participants felt that they did not have further comments to make. Nevertheless, we gathered multiple accounts from the majority of participants which provided a record of the pandemic as it evolved. While open, participant-led research methods do require the researcher to almost take a step back from data collection, there are benefits to be had in terms of encouraging continued participant engagement.

We collected the accounts of 17 participants during the first year of COVID-19. We recruited two practice nurses and 2 practice managers; however, we had intended to recruit a higher number of these 2 participant groups. As a result of these limited numbers, we were unable to compare experiences across the different occupations within the general practice workforce. Nevertheless, we feel that there was a sense of a shared experience across the professions.

## Conclusion

In this article, we have outlined our learning from conducting a rapid research study on the changes made to general practice during the COVID-19 pandemic. We consider our project to be an example of rapid research as the study was established within 4 weeks. Furthermore, it reflects how the research team was required to respond to the changing policy environment of COVID-19 and the changes visited upon general practice service delivery.

A key area of learning in the design and mobilization of rapid research was the advantages of developing a flexible approach to data collection which could respond to participants' situation during the COVID-19 pandemic. The participant-led approach we developed ensured the project could capture the shifts within the COVID-19 pandemic. However, there is a pay-off to be made in that the researcher must be prepared to allow participants the space to contribute as and when they wish. Furthermore, this open approach to data collection was found to encourage multiple dimensions of reflexivity within participants' submissions. This reflexivity is valuable in developing a greater depth to participants' submissions and, in relation, the findings of our study. Rapid research has an important contribution to make to the development and evaluation of policy and interventions. There is much to gain by reflecting on ways in which the research process, from initiation to reporting and dissemination, can be made more efficient and effective to encourage participant engagement and, as a result, strengthen the findings of research. We would like to extend our thanks to the participants within our study who gave their time and enabled us to document the experiences of the general practice workforce during the challenges of the first year of the COVID-19 pandemic.

## Data availability statement

Due to the nature of this research, participants of this study did not agree for their raw data to be shared publicly. There are no data that can be shared.

## Ethics statement

The study received ethical approval from the University of Birmingham Humanities and Social Sciences Ethical Review Committee reference number: ERN_20-0564. The participants provided their written informed consent to participate in this study.

## Author contributions

EB prepared the first draft of the article. JS and LL conceived the study. JS secured ethical approval. EB and LL contributed to data collection. JS, LL, EB, and RF contributed to analysis and interpretation of the results. All authors contributed to editing the article and have approved the final version.

## Funding

This study was supported by the Health Foundation and staff time was provided by the University of Birmingham and the University of Aberdeen. Open access was funded centrally by the University of Birmingham.

## Conflict of interest

Author RF was a practising general practitioner and Senior Policy Fellow at the Health Foundation. Author KS was a practising general practitioner. The remaining authors declare that the research was conducted in the absence of any commercial or financial relationships that could be construed as a potential conflict of interest.

## Publisher's note

All claims expressed in this article are solely those of the authors and do not necessarily represent those of their affiliated organizations, or those of the publisher, the editors and the reviewers. Any product that may be evaluated in this article, or claim that may be made by its manufacturer, is not guaranteed or endorsed by the publisher.
